# Journeying with the Dying—Lessons from Palliative Care Physicians

**DOI:** 10.1007/s41649-024-00321-5

**Published:** 2024-12-14

**Authors:** Lalit Kumar Radha Krishna, Nur Amira Binte Abdul Hamid, Nicole-Ann Lim, Chong Yao Ho, Halah Ibrahim

**Affiliations:** 1 Yong Loo Lin School of Medicine, National University of Singapore, Singapore; 2 Division of Cancer Education, National Cancer Centre Singapore, Singapore; 3 Division of Supportive and Palliative Care, National Cancer Centre Singapore, Singapore; 4 Duke-NUS Medical School, National University of Singapore, Singapore; 5 Centre for Biomedical Ethics, National University of Singapore, Singapore; 6 End of Life Care Centre, Palliative Care Institute Liverpool, University of Liverpool, Liverpool, UK; 7 The Palliative Care Centre for Excellence in Research and Education (PalC), Singapore; 8 Health Data Science, University of Liverpool, Liverpool, UK; 9 Khalifa University College of Medicine and Health Sciences, Abu Dhabi, United Arab Emirates

**Keywords:** Palliative care, Death and dying, Physician–patient relationship, Doctor-patient relationship, Professional identity formation, Personhood

## Abstract

Witnessing suffering and death in palliative care can cause moral distress, emotional exhaustion and maladaptive coping strategies. How sense and meaning is made from these experiences influences how physicians think, feel and act as professionals (professional identity formation or PIF). It also determines how they cope with their roles, care for patients and interact with other professionals. Timely, personalised and appropriate support is key as shaping how these physicians develop and contend with sometimes competing beliefs and roles. The Ring Theory of Personhood (RToP) and the Krishna-Pisupati Model (KPM) offer a means of mapping PIF and thus moulding, coping and meaning making. This study uses the RToP and KPM to explore how caring for end-of-life patients impacts the personhood of palliative care physicians. Semi-structured interviews with a purposive sample of 13 palliative care physicians—eleven females and two males aged between 35 and 50 years—at a cancer specialist centre were conducted. Transcripts of the audio-recorded interviews underwent content and thematic analysis where complementary themes and categories identified were combined to form domains that highlighted the physicians’ key experience of providing end-of-life care. The domains identified were (1) identity formation, (2) conflicts, (3) KPM elements and (4) support systems. Together, results revealed that palliative care physicians are driven by Innate, Individual, Relational and Societal belief systems that create an intertwined professional and personal identity, enabling them to find meaning in their experiences and adapt to present contexts within cultural norms and professional expectations. However, their failure to recognise their need for support when hampered by evolving personal, existential and clinical factors underlines the exigency for ongoing surveillance and a potential role for a RToP-based tool and portfolio system that can detect and direct timely, appropriate support to in-need physicians.

**Supplementary Information:**

The online version contains supplementary material available at 10.1007/s41649-024-00321-5.

## Background

Whilst entrusted with the care of patients at the end of life, consistent exposure to suffering and death can cause moral distress, emotional exhaustion and maladaptive coping strategies in palliative care physicians (Dijxhoorn et al. 2021; Koh et al. 2020). How physicians make sense and meaning of these experiences influences coping, practice and indeed how physicians think, feel and act as professionals (professional identity formation or PIF) (Quah et al. 2023). Timely, personalised and appropriate support is key not only to supporting these physicians but also provides a means of moulding their PIF and shaping their growth as professionals (Radha Krishna et al. 2019, 2020, 2023a, b).

Links between sense and meaning making and shaping PIF and future practice come from a series of studies on a variety of related subjects (Sarraf-Yazdi et al. 2024a, b; Quek et al. 2022; Chiam et al. 2022; Chua et al. 2022b). At the heart of these insights is the notion that how we perceive ‘what makes you, you’ or our personhood guides our self-identity (Sarraf-Yazdi et al. 2024a, b). Specifically, the belief systems that inform and are informed by how our self-concepts of personhood shape our self-identity (Liang et al. 2024). These belief systems are constantly confronting new insights and beliefs drawn from reflections, insights, feedback and how we make sense and meaning of sociocultural, relational, existential, clinical, personal, academic, professional and research experiences that run parallel or contrary to our current beliefs (Wan et al. 2024; Lim et al. 2023). How we integrate these resonate or dissonant belief systems into our regnant belief systems shapes our personhood and changes our PIF (Ibrahim et al. 2024). Thus, supporting sense and meaning making offers a chance to shape how physicians interact, relate, support and care for their patients and colleagues (Ho et al. 2020; Ho et al. 2022; Ngiam et al. 2020; Thompson et al. 2021). Hence, we ask, *‘how does caring for patients at the end-of-life impact the personhood of palliative care physicians?’*

**Fig. 1:**
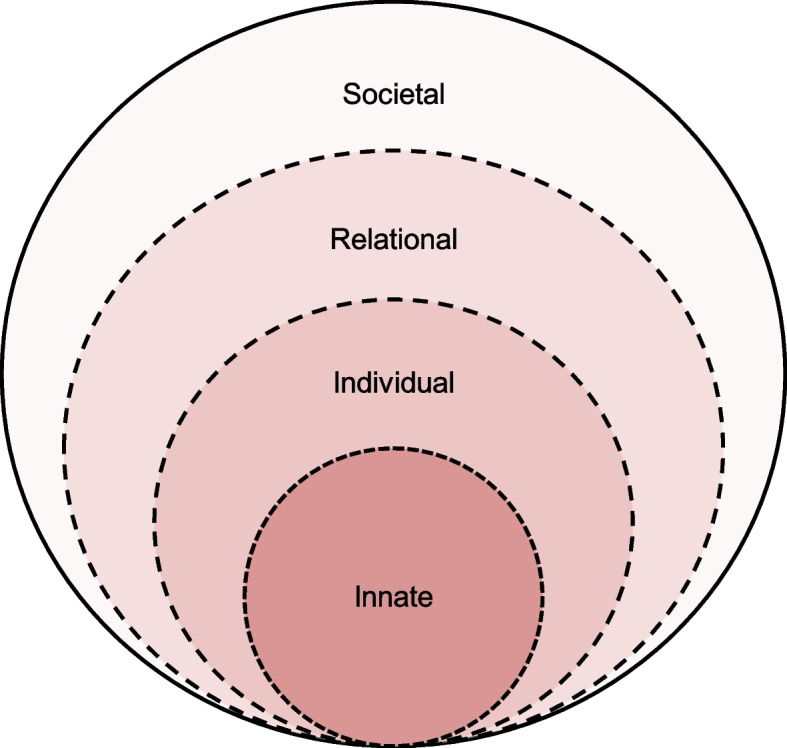
The Ring Theory of Personhood (RToP) (Radha Krishna and Alsuwaigh 2015)

Guided by literature on health professionals’ experiences with terminal patients (Dijxhoorn et al. 2021; Ho et al. 2020; Chan et al. 2021; Kuek et al. 2020), we adopt the Krishna-Pisupati Model (KPM) to map the effects of these experiences on a physician’s personhood. The KPM is built around the Ring Theory of Personhood (RToP) (Ho et al. 2022), which models changes in concepts of personhood perpetuated by shifts in one’s belief systems (Fig. 1). The KPM extends this line of thinking to frame how such changes in belief systems alter self-concepts of identity and bring about changes in personhood to better support physicians.

The RToP proposes that personhood is composed of four rings—Innate, Individual, Relational and Societal, each containing a distinct belief system that informs an individual’s self-concept of identity (Radha Krishna 2014b). The belief system within the Innate Ring is shaped by religious and cultural beliefs, moral values and ethical principles. The belief system within the Individual Ring is informed by autonomous function and individual characteristics. Values governing personal relationships are housed within the Relational Ring, whereas the Societal Ring encompasses the belief system guiding peripheral relationships and societal, professional and legal expectations (Radha Krishna and Alsuwaigh 2015). When new beliefs, values, principles, expectations and considerations (collectively ‘life experiences’) are introduced and clash with existing belief systems, changes in the belief systems lead to changes in self-concepts of personhood and identity, ultimately leading to modifications in thinking and conduct (Ong et al. 2022; Ho et al. 2020; Chan et al. 2021; Kuek et al. 2020).

**Fig. 2:**
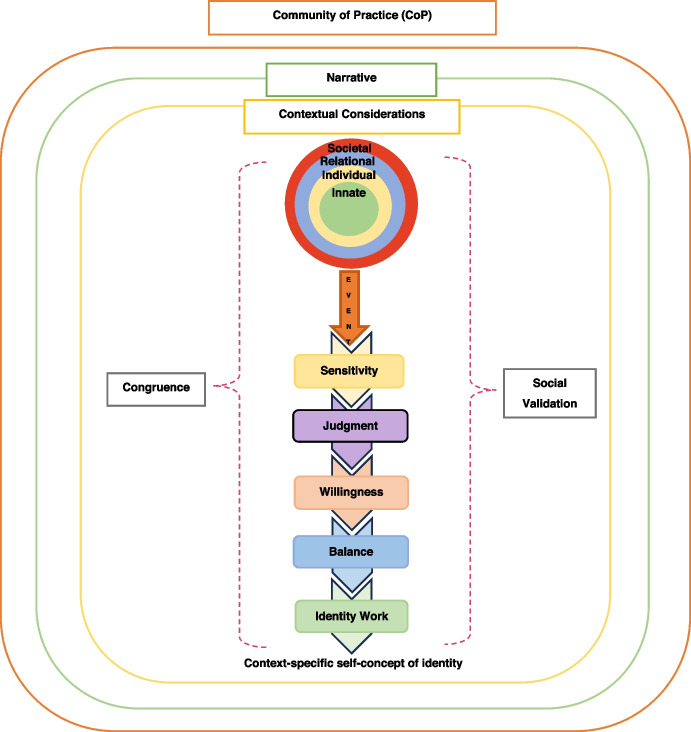
The Krishna-Pisupati Model of professional identity formation (KPM)

The KPM (Teo et al. 2022) helps frame such changes in personhood and identity caused by these new life experiences (Fig. 2). When such experiences reflect and preserve the preexisting belief system, *resonance* in the rings is achieved. A reprioritisation of these resonant beliefs to better fit with the physician’s practical consideration precipitates *synchrony*. In contrast, *dissonance* occurs when new life experiences and prevailing belief system are incongruent. *Disharmony* is born out of dissonance in one ring whilst *dyssynchrony* encompasses dissonance between the rings. The recognition or *sensitivity* to *resonance*, *synchrony*, *disharmony* and/or *dyssynchrony* sees physicians determine if a response is needed (*judgement*) and consider their capacity, ability and motivations (*willingness*) to adapt their identity. Physicians may practice *balance* in prioritising these adaptations to safeguard their overall identity. How they adapt their identity is encapsulated by *identity work*.

## Methods

### Semi-Structured Interviews

It is posited that physicians’ *sensitivity*, *judgement*, *willingness*, *balance* and *identity work* in the face of an event determine how they cope with moral distress. To evaluate this theory, we conducted a series of semi-structured interviews with palliative care physicians at a local tertiary cancer specialist centre to explore the unique lived experiences of clinicians caring for patients at the end of life. Secondary analysis was done of interview transcripts, building on previous work on physician patient boundaries in palliative care (Ho et al. 2023).

### Systematic Evidence-Based Approach (*SEBA*)

**Fig. 3:**
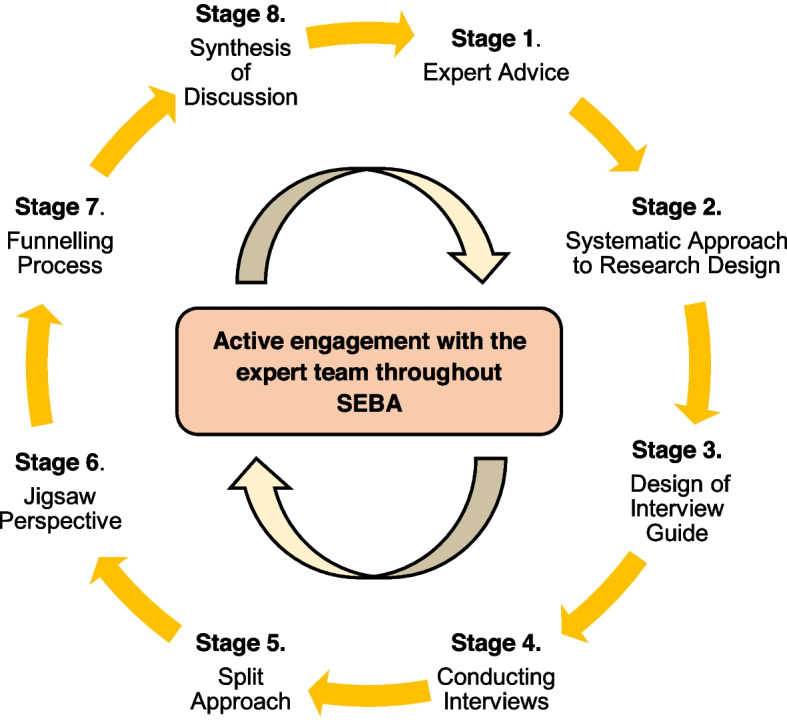
The Systematic Evidence-Based Approach (SEBA) (Ho et al. 2022)

To guide the design of the interview questions, the research team adopted the Systematic Evidence-Based Approach (SEBA) (Fig. 3). Traditionally employed to guide systematic reviews, the SEBA methodology’s constructivist approach (Ng et al. 2020; Bok et al. 2020; Ngiam et al. 2020; Radha Krishna et al. 2020) and relativist lens (Crotty 1998; Ford et al. 2012) are well suited in guiding a structured approach to prospective studies involving context-specific interview scripts. This method is also consistent with regnant theories of sociocultural roots underpinning self-concepts of personhood, belief systems, moral and ethical compass. SEBA’s 6-stage approach (Radha Krishna 2020) was expanded to an 8-stage approach with design of interview guide and the conduct of interviews as shown in Fig. 3 (Ho et al. 2023).

#### Stage 1: Expert Advice

This study included a research team and an expert team (see Online Resource 1). The research team, guided by a senior palliative care physician with experience in qualitative research and use of the SEBA methodology, performed data collection, analysis and manuscript writing. The expert team, comprised of medical educators, psychologists and clinicians from a cancer centre, a palliative care institute and regional medical schools, oversaw the methodology to enhance accountability, trustworthiness and reproducibility.

i. Reflexivity

The lead author is a senior palliative care physician who was able to understand nuances of the specialty, thereby strengthening the rigour of the study (Wilson et al. 2022). To minimise social desirability bias (Andriyani et al. 2021), we recruited two trained interviewers who did not have working relationships with the participants (McNair et al. 2008). In maintaining reflexivity in data interpretation, the authors consulted members of the expert and research teams to attenuate the impact of personal experiences and biases on the study.

#### Stage 2: Systematic Approach to Research Design

The expert and research teams adopted a systematic approach to review the current literature (Radha Krishna 2013, 2014b) on the impact of caring for the dying on health professionals (Huang et al. 2022; Quek et al. 2022; Ho et al. 2022; Ong et al. 2022; Vig et al. 2021; Radha Krishna 2014a, 2011). These series of reviews, commentaries and studies have since been published (Radha Krishna and Kwek 2015; Radha Krishna and Ho 2015; Radha Krishna et al. 2017).

#### Stage 3: Design of Interview Guide

The results of Stage 2 guided the design of semi-structured interview questions meant to elicit the impact of lived experiences of palliative care physicians on their belief systems around the four domains of the RToP (Creswell et al. 2017). Through a modified Delphi process, palliative care experts and qualitative researchers reviewed and revised the interview guide (see Online Resource 2).

#### Stage 4: Conducting Interviews

Purposeful recruitment guided the distribution of email invitations containing participant information sheets and study details to all palliative care physicians at a local cancer specialist centre. The invitations emphasised participant anonymity and the right to withdraw from the study without prejudice. 15 invitations were sent. 13 were accepted and 2 did not reply due to illness. After obtaining verbal and written consent, two researchers conducted audio-recorded interviews on an institutionally secured Zoom video-conferencing platform between July and August 2021. The interviews were carried out in vacant offices to ensure privacy. Each interview lasted approximately 45 min and data saturation was achieved after nine sessions. An additional 4 interviews were continued to confirm data saturation. The recordings were stored in a password-encrypted device, transcribed verbatim via the NVivo 12 Software, and anonymised. Member checking was performed with each participant. Ethics approval was obtained from an institutional review board. All methods were carried out in accordance with relevant guidelines and regulations.

#### Stage 5: Split Approach

The adapted SEBA methodology employed the concurrent application of Hsieh and Shannon (Hsieh and Shannon 2005)’s approach to directed content analysis and Braun and Clarke (Braun and Clarke 2006)’s thematic analysis. This combined methodology mitigated the limitations of each data analysis method and helped to facilitate the understanding of terminology among various team members. For example, content analysis could account for contradictory data and negative results often omitted within thematic analyses (Chan et al. 2021; Ng et al. 2020; Nowell et al. 2017).

i. Thematic Analysis

Two members of the research team adopted Braun and Clarke (Braun and Clarke 2006)’s approach to thematic analysis to construct codes from the surface meaning of the transcripts. The members then discussed the codes and categorised them into corresponding groups, employing Sandelowski and Barroso (Sandelowski and Barroso 2006)’s approach to ‘negotiated consensual validation’ to determine the final codes, themes and subthemes. Discrepancies were resolved through consensus. Code books were maintained to identify assumptions and enable auditing (Braun and Clarke 2019).

ii. Directed Content Analysis

Concurrently, two other research team members employed Hsieh and Shannon (Hsieh and Shannon 2005)’s approach to directed content analysis to independently analyse the transcripts. Deductive content analysis was conducted using codes (Mayring 2004; Kibiswa 2019) drawn from Kuek et al. (Kuek et al. 2020)’s *‘The impact of caring for dying patients in intensive care units on a physician’s personhood: a systematic scoping review’* and Thompson et al. (Thompson et al. 2021)’s *‘Doing healthcare at end-of-life: Identity tensions, negotiations, and conflicts’.* Data uncaptured by a priori codes were assigned a new code (Hsieh and Shannon 2005). Through ‘negotiated consensual validation’, we achieved consensus on the final coding scheme (Elo and Kyngäs 2008).

#### Stage 6: Jigsaw Perspective

The Jigsaw Perspective brought together overlapping elements of the identified categories to form themes and subthemes (France et al. 2019). Reciprocal translation was employed to determine if the themes and categories could be used interchangeably.

#### Stage 7: Funnelling Process

In the Funnelling Process (Noblit and Hare 1988), the themes and subthemes were systematically compared and juxtaposed to form domains that informed the discussion in Stage 8 (Radha Krishna 2020).

## Results

Participants included eleven female and two male physicians, aged between 35 and 50 years (mean 40 years), with an average of 8 years of experience as specialist palliative care physicians. The four domains identified were (1) identity formation, (2) conflicts, (3) elements of the KPM and (4) support systems. Each of the domains is explained in detail below. There is no difference in the themes and subthemes in analysis across age and gender as specialists underwent the same training and similar experiences.

### Domain 1: Identity Formation

In caring for patients at the end of life, physicians experienced significant shifts in the prevailing personal principles that guided their practice and informed their moral and ethical compass—resulting in changes in how they perceived their personhood and identities. Anchoring focus on the changes in their personal principles also provided a context-sensitive and individualised perspective of how elements of personal principles were weighed and balanced. These changes were captured in each of the four rings of the RToP as described below.

#### Societal Ring

Physicians entered the palliative care field advocating for holistic, patient-centred care that extended beyond physical symptom management. These values, combined with the notion of journeying with the patients to the end, underpinned how palliative care physicians viewed and practiced their roles:

The fact that it’s so multi-dimensional. It’s about patients and their lives, them as human beings… To me, it was science together with the patient, as a human being, as a person, that person-to-person connection. Not just sole scientific stuff, but also the psychology of how illness impacts the whole family unit and the person. How do I fit in there? What are my strengths as a professional or as a human? What gifts can I bring to the people? (P3)

You can really treat a patient holistically and really get to know their whole family. You really journey with them as a whole right till the end. (P1)

Despite their initial intentions, the physicians recognised the limitations of their role. This allayed some of the psycho-emotional burden of their early expectations:

I used to want to do a lot for all my patients and it’s not that I don’t do that now, but I also recognize that maybe I’m not the person to meet all their needs. Maybe they need their family, and they need other people. (P11)

When I was even less experienced in my practice, maybe we were a bit more idealistic. We wanted to solve every problem for them, including things like finances, resolving family conflicts. But as I got more and more years in medicine, I understand that that is no longer possible… As much as I want to do everything, we can’t… there are other people out there that may be better equipped. Those are things that have led me to scale back on some of my roles. (P10)

#### Relational Ring

Through prolonged experiences with terminally ill patients and their families, physicians prioritised building and maintaining close personal familial relationships to balance between work and home:

In the end, it’s only the family that matters. This is what we have learnt seeing patients. It’s not how many houses you own. It’s not how many cars you own... In the end, all we end up with is family, or the lack of family. (P9)

Of course, I fear to experience death in my own family, and already when it’s with my own patients, it can get so bad. It always at the back of my mind… That’s why I just concentrate on spending quality time with them. (P3)

#### Individual Ring

Over time, physicians exhibited shifting personal philosophies as they reflected on their experiences:

It also helps me reflect about myself. Over the years, we have to care for patients who are our seniors… It helps me reflect on what I would do [if I were them] and how I would react to certain things… And it also helps me to be more thankful for where I am now at this point in time. (P11)

You value what you have more… to be more keenly aware… Not so much of how blessed I am, but how bad the world. It does definitely, these things combined, inspire me to do more for my patients, to be the best palliative care physician that I can be. It’s inspiring, or it’s at least motivating. (P10)

#### Innate Ring

Caring for the dying reinforced spiritual beliefs for physicians who sought solace in their faiths. A strengthened sense of spirituality served as a safeguard against moral distress:

I do pray, and I go to church… It helps you to make sense and frame your living, especially when you see a lot of suffering around you. I think it helps you make sense of that suffering. I would say I never get distressed because I think I can make sense of why things are like that. (P13)

I guess it has deepened all these beliefs… That we should invest in things that are eternal, with our decisions that we make. These are some of the things that I suppose strengthened what is taught on the pulpit. But when you see it lifted up, not only through yourself, but because of many lives that you see, the regrets people have, the things that they valued throughout their lives, that strengthened those teachings and beliefs. (P7)

### Domain 2: Conflicts

The physicians reported conflicts between their roles and their personal principles within the rings (*disharmony*) and between the rings (*dyssynchrony*) of the RToP.

#### Disharmony Within Rings

Physicians experienced *disharmony* when new experiences in caring for the dying clashed with their prevailing belief systems within each ring of the RToP (Table 1).

**Table 1:** Sources of disharmony within rings of the RToP

Disharmony within rings		Supporting quotes
Societal Ring	Helplessness as a physician to provide for patients	*‘I think it’s the helplessness that as a physician that’s quite heart-breaking. In those days, I still remember feeling quite guilty because we couldn’t save our patient, and he was so young, he didn’t do nothing to have this.’ (P11)* *‘If the patient’s very symptomatic, sometimes it makes you wonder, what else can you do?… Then sometimes with the caregivers it’s like, if they have unresolved issues or they’re still coming to terms with things, then again, I wonder, what else can we do to help them?’ (P8)*
	Burn out	*‘I felt fatigue… I did come to a point where I was starting to not really care about what happens at work.’ (P8)* *‘In terms of disinterest in work and feeling stressed. Tiredness, fatigue, definitely.’ (P4)*
Relational Ring	Negative impact on relationships with loved ones	*‘After a while, where you’re dealing with such heavy stuff and you go out there and you listen to your friends’ frivolous conversations… you really don’t want to be there anyway. And so, I think for some of the junior staff, one of the potential challenges they might have is actually, they start feeling that what they’re doing is so intense and so heavy and so important that it’s difficult to connect with other people out there.’ (P4)* *‘Initially, I brought work home. I realised over the years that my family cannot take all these sad stories… It actually distresses them’ (P3)*
Individual Ring	Challenged personal principles	*‘I feel we should be treating people equally, in whichever way is appropriate for them, or just give them the best care we can. It doesn’t matter who they are [but] there’s so much social inequality. Even if you talk about wanting to treat a rich patient as well as I treat a poor patient, the options to a rich person is so much different.’ (P9)*
Innate Ring	Challenged spiritual beliefs	*‘You feel very distant, and you can’t be bothered to pray, like prayer is also no use.’ (P3)* *‘That leads some to some spiritual questioning… there are times when I do see … another person that is a mutual Christian, or even a preacher, fall away last minute because of suffering. And you wonder, how can this happen?’ (P7)*

#### Dyssynchrony Between Rings

Instances of *dyssynchrony* were evident as physicians alluded to conflicts between the rings of the RToP (Table 2).

**Table 2:** Sources of dyssynchrony between rings of the RToP

Dyssynchrony between rings	Conflict	Supporting quotes
Societal vs Relational Rings	Some physicians struggled to navigate their relationships with patients, revealing a blurring of the physician–patient line as they developed personal relationships. This, at times, threatened professional boundaries and impaired medical judgement and was not always in the patient’s best interests	*‘I got a bit too close to a patient… What happened to him emotionally affected me. And I started questioning my decision metric because I didn’t know whether I was deciding as a doctor or as a friend. And so, it became very tricky. So, I started questioning some of the things I was trying to do for him or the strings I was pulling.’ (P5)*
	For some physicians, forming strong, emotional relationships with patients led to profound grief upon the patient’s death	*‘Sometimes I get a bit soft. I tell myself she’s already lost so much, if it really is a comfort to her, then offer my friendship…. We continued messaging each other and we’d almost chat like friends. I know it was very blurred and sometimes I was a bit confused. At the end of the day, she suddenly died, very unexpectedly…. I felt that if I had maintained some boundaries, I wouldn’t be so hurt, because I grieved for her… In fact, quite long. It’s more so than any other patient of mine ever. I still feel that for me, at least, in my journey as a professional, such experiences also help me gain wisdom about my limits, like why boundaries are there.’ (P3)*
	The emotional weight of coping with the death of patients led to the establishment of strict physician–patient boundaries	*‘I usually tend to draw quite a clear distinction between my professional and also my personal emotions. Some might say that means you’re not doing enough. But I think, to me, it’s something that I’ve always been able and so far, I’ve never encountered a case whereby I was too emotionally traumatized.’ (P6)*
Societal vs Individual Rings	Physicians highlighted the strain on their personal values, beliefs, and principles from the demands of work obligations	*‘In palliative care, because it is whole person care, it calls on all of our resources as a person… it could also be very draining if we haven’t learnt how to replenish ourselves after either sharing yourself or seeing suffering… The question is, after you are drained, how do you replenish that cup? Because for every patient who we lose, every patient who dies, we lose a bit of ourselves and we grieve for every patient.’ (P9)*
Societal vs Innate Rings	Issues of spiritual distress surfaced as physicians found themselves grappling between their wider role as a professional and their innate role as a religious practitioner	*‘I’m her doctor, yet talking about such intimate things like spirituality put me in this very unique position where I help to address distress. But then how do you address such spiritual distress? How do you separate your identity or your rules and what you’re supposed to do? I was struggling in terms of professionally, like, where am I? How am I going to help her? Am I supposed to do this? I’m here to listen to her. How do I also minister to her? Do I minister to her? Well, I would have if I was a human being, but as a professional, do I minister to her?’ (P3)*
	For other physicians, their willingness to address a patient’s spiritual distress was hindered by their lack of training in spiritual care and knowledge of the major religions	*‘I’m a Christian… what I’m struggling with is actually to be able to apply it to my patient care. Yes, because as a physician there is some professional boundaries. But at the same time, you want to use it to your advantage if it’s to identify a problem or, rather, an area in which you can potentially help the patient. Yes, so I’ll be completely honest, I have no experience in this.’ (P7)*

### Domain 3: Elements of the KPM

Embedded in the experience of caring for the terminally ill were the elements of the KPM and their impact on the thinking, decision-making, actions and conduct of physicians. Here, we focus on the issue of burnout that was commonly evoked by physicians during the interviews. The elements identified were sensitivity to burnout, judgement that it needs to be addressed, willingness to address burnout, balance with self-care as well as reflection to break the cycle of burnout.

#### Sensitivity and Judgement

Most physicians displayed *sensitivity* in recognising the signs of burnout. These included physical and mental exhaustion, emotional detachment, irritability, loss of motivation and decreased work satisfaction. Concurrently, physicians’ *judgement* emphasised the importance of addressing these symptoms.

You realize it when you start feeling a bit snappish and angsty against certain patients. So, that’s when you realize that this is not usually how you should react to these kind of issues. It is about time to take it easy and take a break. (P1)

Dreading to go to work is a big sign for me. It’s not just physical, there’s multidimensions … Physically, you might get some symptoms, like no sleep, or just low attention, can’t concentrate, very tired and fatigued, or you get psychological symptoms, like I might feel very anxious, edgy, irritable, especially angry. Spiritually, sometimes I get disconnected. (P3)

If left unattended, the signs of burnout became evident to colleagues:

I have seen burnout in other colleagues… They get emotionally distraught. Their tempers will flare. I think the problem with burnout is it affects other people. That it hits the people surrounding… And therefore, it makes them increasingly tired also. (P5)

Their moods are always not that good. I mean, you can see it in them. They just look like depressed all the time. I haven’t really seen it manifested in like medical mistakes or medical errors so far. But mainly the way they communicate, and their general behaviour. (P10)

#### Willingness and Balance

Upon recognising the *disharmony* and/or *dyssynchrony* in burnout and making the *judgemen*t that there is a need to address them, physicians showed *willingness* and *balance* in prioritising self-care to preserve their overall identity:

I think you must understand that you have a responsibility to care for yourself. So, some people propagate these things like self-care, taking specific time out to be mindful… I think that’s actually important. (P13)

So now I take frequent breaks, so I make sure I always have a scheduled break just to reset and recharge. (P8)

#### Reflection

Over time, physicians found themselves engaging in self-reflection and recognising the importance of reflective practice in breaking the cycle of burnout:

I think the important thing is it always comes with insight, because with insight you can have action… We are empowered to say that… it’s a bit too much now or getting a bit too deep. (P13)

Medicine can be very emotionally or mentally draining. And therefore, it is about protective mechanisms. But different people work differently with different protective mechanisms. So, you’ve got to figure it out and figure it out fast before you go into cycle of burnout. (P5)

### Domain 4: Support Systems

In abating *disharmony* and *dyssynchrony*, physicians often turned to their support systems, namely social and institutional support.

#### Social Support

Most physicians relied on social support from colleagues to help tackle issues of *disharmony* and *dyssynchrony*. They confided in fellow physicians to seek advice and express grievances in their unique shared experiences. This outlet served as a buffer against occupational stress and burnout:

The fact that we have close teams that we can bounce off our frustrations with… Sometimes, when you see such heavy things going on a daily basis, it’s very difficult to go out there and have a meal with your non-medical friends and talk about these things. Because they wouldn’t understand. It’s a bit too heavy for normal meal conversation. And so, you actually rely on people within the system that understand. (P4)

I feel that a lot of times, you’ll find that your close colleagues are the ones that will pick you up when you need support. And I think that works quite well. (P1).

#### Institutional Support

Physicians appreciated institutional efforts to destigmatise psychological challenges and promote the use of mental health resources, with most preferring debrief sessions facilitated by an external psychiatrist. By providing a safe and private space to address workplace trauma and conflicts, these sessions encouraged self-reflection and healthy coping mechanisms.

One thing that was very good that was done many years back by the department was this psychiatrist who would check on us, very closed-door sessions. And in it, you could say anything and everything. It could be about your colleague, it could be about your boss, about work. You could be sharing about a case, and everything. That psychiatrist was very good. And it helped. (P5)

We have an opportunity to have direct contact with a retired psychologist, and she gives us a regular debrief if we want. So, that’s a good way, I feel, to self-care, because you take that active step to process that trauma that you might have from the patient. (P13)

However, with the discontinuation of these psychiatrist-mediated sessions, other support initiatives, including music therapy and structured staff support and workshops, were considered inadequate and even counterproductive.

[The psychiatrist] is not doing any more. So now, we have some team support stuff, and you have music therapy. But I don’t find it very useful. Therapy, I do find very useful. (P5)

The support has to be genuine. I think, unfortunately, the staff support, the way it’s being done nowadays, in our department, I think the intent is good… but unfortunately doesn’t feel very genuine to me. So, it feels a bit superficial, like we’re just having this because we’re [palliative physicians] so we need to have staff support. (P2)

## Discussion

### Stage 8: Synthesis of Discussion

Our study illustrates how work in the palliative care setting significantly impacts all aspects of a specialist’s life. Boundaries between the personal and professional realms are often blurred, as work issues encroach on family and personal time. As we note from the responses, work experiences also consistently challenge a physician’s belief system—witnessing suffering and death can lead to questioning of spiritual beliefs, as well as feelings of inadequacy and helplessness. Navigating this dissonance requires physicians to balance between relying on their guiding belief systems and confronting, discussing and adapting them with time and circumstance. However, physicians are not always successful, particularly when efforts to find balance and make effective meaning from their experiences are hampered by evolving personal, existential and clinical factors. Perhaps more concerning, physicians often do not recognise their need for support. When inadequately supported, it was found that some palliative care physicians erect rigid demarcations between their professional and personal lives as a form of self-preservation. Such a boundaried sense of self often hinders self-regulation and predisposes to occupational stress, anxiety, burnout, loss of meaning and attrition. It further limits help resulting in negative impacts on patient care. Understanding these responses is key to supporting palliative care specialists and trainees. 

This brings forth the importance of multifaceted support to be rendered to palliative care specialist as they continue to work in this field.

Institutional and team support are thereby essential. Often, it is the team that recognises growing distress and intervenes on behalf of the struggling physician. Within the context of a nurturing department and team culture, a combination of peer debriefs, existential and team-based support and integration of reflective practice appears to mitigate maladaptation. Whilst these findings are not new (Zambrano et al. 2012; Mathews et al. 2021), it is clear in this study that support remains insufficient or ineffective and more work can be done to evaluate and improve existing programmes. A combination of counselling services, guided debriefs, personalised psychological care and facilitated support in a safe setting is required and cannot be implemented without committed and radical investment from top-brass in the institution. It is notable that palliative care physicians find more value in private and protected time for reflection and discussion, facilitated by an external and independent professional than through general wellbeing or team-building activities that are seen as ineffective surface level interventions.

Key and unique to this study is the role of religious and spiritual beliefs, strong familial ties and close collegial relationships which play key roles in ‘recovery’ and mitigating burnout. For this reason, institutions must invest in physicians holistically allowing for sufficient time off for physicians to strengthen existing relationships, reflect and make meaning of their experiences. Spiritual care can assist in abating existential distress and help physicians navigate difficult conversations on spirituality with patients. Palliative care physicians also require time off work to recover and reflect upon their emotions to process the helplessness and grief they often experience after the death of their patients. Akin to the concept of total pain (Clark 1999) for palliative care patients where suffering cannot be addressed incompletely by only focusing on the physical, psychological, social and spiritual domains, burnout cannot be addressed in physicians by only focusing on the societal ring of the RtoP.

This study also demonstrates that effective coping with death and dying requires reflexive construction or the integration of perspectives and reflections (Southall 2013; Kovecses 1987). The notion of reflexive construction maps the influence of physicians’ personal experiences, changing personal principles and narratives on identity formation (El Nawaw et al. 2012; Chua et al. 2022a) and supports the need for supervised immersion into the clinical setting, longitudinal mentored support systems, guided reflections, debriefs and individualised support mechanisms. It also calls for a nurturing learning environment replete with clearly defined expectations, roles, and responsibilities and a code of conduct for all stakeholders. This must include structured training for physicians that extends to access to personalised counselling, mentoring, coaching and supervision, guided reflective practice and self-care programs. Lastly, underlining the longitudinal and often unpredictable nature of psycho-emotional distress, reflexive construction underscores the need for ongoing surveillance of palliative care physicians, suggesting a role for portfolios. These portfolios should include a RToP-based tool to evaluate physicians over time to direct support and follow-up of the physicians’ reflexive construction of their professional identities.

### Limitations

The findings of this study may be limited by its unique use of the RToP to guide the interviews and analysis. Whilst concurrent use of thematic and directed content analysis by independent coders sought to minimise bias in the interpretation of the data, there may be some implicit bias persistent in the analysis as individual interpretation of data may be affected by researchers’ own personal and professional experiences. Whilst the data was compared to existing papers and there is congruence in results, there may be still limits to generalisability due to the small single-site study. Furthermore, due to social desirability bias, interviewees may be hesitant to share negative experiences with interviewers even though there was anonymity and no dependent relationship between interviewers and interviewees.

## Conclusion

Our findings shed light on how palliative care physicians are impacted by their work with death and dying, thereby affecting both their professional and personal identities which are deeply intertwined. For this reason, interventions targeting professional support alone are insufficient and further studies can be done to design and utilise holisticprovide.

## Supplementary Information

Below is the link to the electronic supplementary material.

Supplementary file1 **Online Resource 1.** Demographics of Expert and Research Team Members (DOCX 4.52 MB)

Supplementary file2 **Online Resource 2.** Interview Guide (DOCX 7.77 MB)

Supplementary file3 **Online Resource 3.** Demographics of Participants (DOCX 13.6 KB)

Supplementary file4 **Online Resource 4.** COREQ Checklist (PDF 481 KB)

## Abbreviations

KPM: Krishna-Pisupati Model of professional identity formation
RToP: Ring Theory of Personhood
SEBA: Systematic Evidence-Based Approach

## Declarations

### Materials and/or Code Availability

The authors confirm that the data supporting the findings of this study are available within the article and its supplementary materials.
