# Supplementary material for: Journeying with the Dying—Lessons from Palliative Care Physicians
Source: Asian Bioeth Rev. 2024 Dec 14;17(3):591–613. doi: 10.1007/s41649-024-00321-5 (PMC12304369; doi:10.1007/s41649-024-00321-5)
Supplement: Supplementary file 1 — Supplementary file1 Online Resource 1. Demographics of Expert and Research Team Members (DOCX 4.52 MB) [file 41649_2024_321_MOESM1_ESM.docx]

**Online Resource 1.** Demographics of Expert and Research Team Members

| Team Member | Occupation | Qualification | Specialty |
| --- | --- | --- | --- |
| *Expert Team* | | | |
| Member 1 | Psychologist | PhD | Medical Education |
| Member 2 | Psychologist | PhD | Medical Education |
| Member 3 | Medical Librarian | PhD | Medical Education |
| Member 4 | Health Informatics | PhD | Health Services Research |
| Member 5 | Pharmacist | Masters (Med Edu) | Medical Education |
| *Research Team* | | | |
| Lead Author | Physician | PhD, MD, MBChB | Palliative Care |
| Member 1 | Physician | MBBS | Palliative Care |
| Member 2 | Physician | MBBS | Palliative Care |
| Member 3 | Physician | MBBS, Masters (Med Edu) | Infectious Diseases |
| Member 4 | Physician | MBBS, Masters (Med Edu) | Rheumatology |
| Research Team Members  5 to 12 | Medical Students | Nil | Research Mentees |
| Research Team Members  13 and 14 | Research Assistant | Nil | Research Assistants |
| Interviewer 1 | Research Manager | Master (Med Hum) | Medical Humanities and Education Researcher |
| Interviewer 2 | Research Assistant | Psychology | Clinical Psychology |
