# Supplementary material for: Journeying with the Dying—Lessons from Palliative Care Physicians
Source: Asian Bioeth Rev. 2024 Dec 14;17(3):591–613. doi: 10.1007/s41649-024-00321-5 (PMC12304369; doi:10.1007/s41649-024-00321-5)
Supplement: Supplementary file 3 — Supplementary file3 Online Resource 3. Demographics of Participants (DOCX 13.6 KB) [file 41649_2024_321_MOESM3_ESM.docx]

| ID | Work Experience (Years) | | Gender |
| --- | --- | --- | --- |
|  | As a doctor | As a Palliative Care Physician |  |
| P1 | 14 | 3 | F |
| P2 | 9 | 3 | F |
| P3 | 15 | 8 | F |
| P4 | 24 | 12 | F |
| P5 | 12 | 5 | M |
| P6 | 6 | 2 | M |
| P7 | 17 | 11 | F |
| P8 | 15 | 4 | F |
| P9 | 48 | 30< | F |
| P10 | 10 | 2 | M |
| P11 | 14 | 8 | F |
| P12 | 25 | 7 | F |
| P13 | 17 | 9 | F |
